# 626 Retaining Burn ICU Heroes: Strategies for Staff Empowerment

**DOI:** 10.1093/jbcr/iraf019.255

**Published:** 2025-04-01

**Authors:** Anna Ehoff, Liane Nguyen, Catrina Cullen, Nicole Kopari

**Affiliations:** Leon S. Peters Burn Center; Leon S. Peters Burn Center; Leon S. Peters Burn Center; UCSF Fresno Leon S. Peters Burn Center

## Abstract

**Introduction:**

Multiple ICUs across the nation are struggling with staff turnover. The Burn ICU was experiencing high turnover due to staff burnout, leadership gaps, workplace incivility, and a low applicant pool. Burnout is caused by short staffing, staff not feeling heard, inconsistent rounding with leadership, lack of information, and decreased recognition. We aimed to build a healthy work environment by making small changes to support staff.

**Methods:**

New processes were initiated for the staff to promote staff retention. This included one-on-one staff rounding/interviews every quarter, utilizing stress first aid assessments, monthly staff meetings to increase communication, and enhancing staff recognition through the Shout Out recognition board and an Employee of the Month program. The one-on-one staff rounding tool comprised five open-ended questions and included the stress first aid assessment, which allowed staff to build their resiliency skills. Monthly staff meetings were initiated to keep staff informed of hospital and unit-based changes. During this monthly meeting, we have recognition for our employee of the month, new staff, and shout-out recognitions.

**Results:**

Our turnover rate for January 2023 to December 2023 was 23.08%. We initiated our methods in January 2024. Our post-implementation results showed the January 2024 to July 2024 turnover rate was 10.9%

**Conclusions:**

Quarterly rounding and the stress first aid tool ensure staff feel heard. This tool gives leaders a tool to assist staff with resilience and teach self-care. It educates people on improving resiliency and gaining skills to help with positive coping measures. Recognition with shout-outs and employee of the month shows staff that they have a purpose in what they do. The staff meetings keep staff informed and promote and foster a culture of learning, recognition, and teamwork.

**Applicability of Research to Practice:**

Staff turnover is high across the nation, and training new staff is expensive. As we recover from the COVID-19 pandemic, now more than ever, we as leaders must make our staff feel safe, valued, and important to our facilities.

**Funding for the Study:**

N/A

